# Effects of poverty on mental health in the UK working-age population: causal analyses of the UK Household Longitudinal Study

**DOI:** 10.1093/ije/dyac226

**Published:** 2022-12-08

**Authors:** Rachel M Thomson, Daniel Kopasker, Alastair Leyland, Anna Pearce, S Vittal Katikireddi

**Affiliations:** MRC/CSO Social and Public Health Sciences Unit, University of Glasgow, Glasgow, UK; MRC/CSO Social and Public Health Sciences Unit, University of Glasgow, Glasgow, UK; MRC/CSO Social and Public Health Sciences Unit, University of Glasgow, Glasgow, UK; MRC/CSO Social and Public Health Sciences Unit, University of Glasgow, Glasgow, UK; MRC/CSO Social and Public Health Sciences Unit, University of Glasgow, Glasgow, UK

**Keywords:** Mental health, poverty, income, causal methods, health inequalities, depression

## Abstract

**Background:**

Addressing poverty through taxation or welfare policies is likely important for public mental health; however, few studies assess poverty’s effects using causal epidemiology. We estimated the effect of poverty on mental health.

**Methods:**

We used data on working-age adults (25–64 years) from nine waves of the UK Household Longitudinal Survey (2009–19; *n *= 45 497/observations = 202 207 following multiple imputation). We defined poverty as a household equivalized income <60% median, and the outcome likely common mental disorder (CMD) as a General Health Questionnaire-12 score ≥4. We used double-robust marginal structural modelling with inverse probability of treatment weights to generate absolute and relative effects. Supplementary analyses separated transitions into/out of poverty, and stratified by gender, education, and age. We quantified potential impact through population attributable fractions (PAFs) with bootstrapped standard errors.

**Results:**

Good balance of confounders was achieved between exposure groups, with 45 830 observations (22.65%) reporting poverty. The absolute effect of poverty on CMD prevalence was 2.15% [%-point change; 95% confidence interval (CI) 1.45, 2.84]; prevalence in those unexposed was 20.59% (95% CI 20.29%, 20.88%), and the odds ratio was 1.17 (95% CI 1.12, 1.24). There was a larger absolute effect for transitions into poverty [2.46% (95% CI 1.56, 3.36)] than transitions out of poverty [–1.49% (95% CI –2.46, –0.53)]. Effects were also slightly larger in women than men [2.34% (95% CI 1.41, 3.26) versus 1.73% (95% CI 0.72, 2.74)]. The PAF for moving into poverty was 6.34% (95% CI 4.23, 8.45).

**Conclusions:**

PAFs derived from our causal estimates suggest moves into poverty account for just over 6% of the burden of CMD in the UK working-age population, with larger effects in women.

Key Messages

- Moving below the poverty line increases prevalence of common mental health problems in UK working-age adults by 2.5%, with an odds ratio of 1.21.
- New poverty explains 6.3% of the current burden of poor mental health in the UK, if these estimates are unbiased.
- The detrimental impacts of transitions into poverty are larger than the positive effects of moves out of poverty.
- The mental health of women is potentially more sensitive to poverty.

## Introduction

Income levels are widely thought to be a key determinant of mental health and wellbeing, and in particular a key driver of mental health inequalities.^1^ This is evidenced on a macro-level, where population mental health appears sensitive to macroeconomic events such as recessions and the policy decisions which follow them.^2^^,^^3^ It also appears true at an individual level, where income changes have been linked to changes in mental health, particularly where individuals move into poverty (i.e. below a level of income considered necessary to maintain an adequate standard of living).^4^^,^^5^

However, there are methodological challenges in overcoming issues of bias and confounding when considering the income-health relationship, particularly the possibility of reverse causation due to health selection (where those with poor mental health experience falls in income).^6^ This, and heterogeneity in the evidence base, has raised questions around the size of any health benefit conferred by income changes,^5^ and even challenges around whether an effect exists at all.^7^ For policy makers to make informed decisions on whether polices are likely to improve population mental health, more accurate estimates of these relationships and how they may vary across different groups are required.^8^

Some studies have attempted to reduce potential bias by excluding those with pre-existing mental health problems to address reverse causation, or by using approaches such as fixed-effects regression which account for time-invariant confounding factors (for example, ethnicity or early life socioeconomic circumstances).^4^^,^^9^^,^^10^ However, accounting for time-varying confounding is more problematic, particularly for confounders which also lie on the causal pathway between the exposure and outcome.^11^ In these circumstances, traditional statistical methods which simply control for such variables block part of the causal pathway of interest, potentially leading to a biased estimation of the true causal effect.^12^

Methods which explicitly incorporate potential causal pathways may be more appropriate to investigate a relationship of this complexity in observational data.^13^ We used one such method, marginal structural modelling, to estimate the effect of poverty on mental health outcomes in the working-age population, and to investigate potential effect modification by gender, education, and age.

## Methods

### Dataset and population

We used data from nine waves of the UK Household Longitudinal Study (UKHLS, also known as Understanding Society), which includes around 40 000 households randomly sampled from the community-dwelling population from 2009 to 2019.^14^ Data were collected annually on those aged 16 years or over within included households, via in-person interview or an online self-completion questionnaire. Analysis was restricted to working-age adults aged 25–64 years to allow educational attainment to be used as a relatively stable measure of adult socioeconomic position.

### Exposure

Data on monthly and annual household income and its source (i.e. unearned versus earned income) were collected in all UKHLS waves. We generated a binary variable indicating whether individuals were living in a household which was above or below the poverty line at each time point, defined as household equivalized income <60% of that year’s median after housing costs. Our use of the after-housing costs measure provided the closest estimate possible to the true total disposable income and resources available to households once they had paid their necessary non-discretionary costs, and is in keeping with the approach taken by the UK Social Metrics Commission.^15^

### Outcome

Prevalence of mental health problems was assessed using the General Health Questionnaire-12 (GHQ-12), a validated screening tool for mental health problems used widely in epidemiological research.^16^ It generates a score between 0 and 12, with four or above indicating a strong likelihood of common mental disorder (CMD), often referred to as ‘caseness’.^17^

### Causal framework

**Figure 1:**
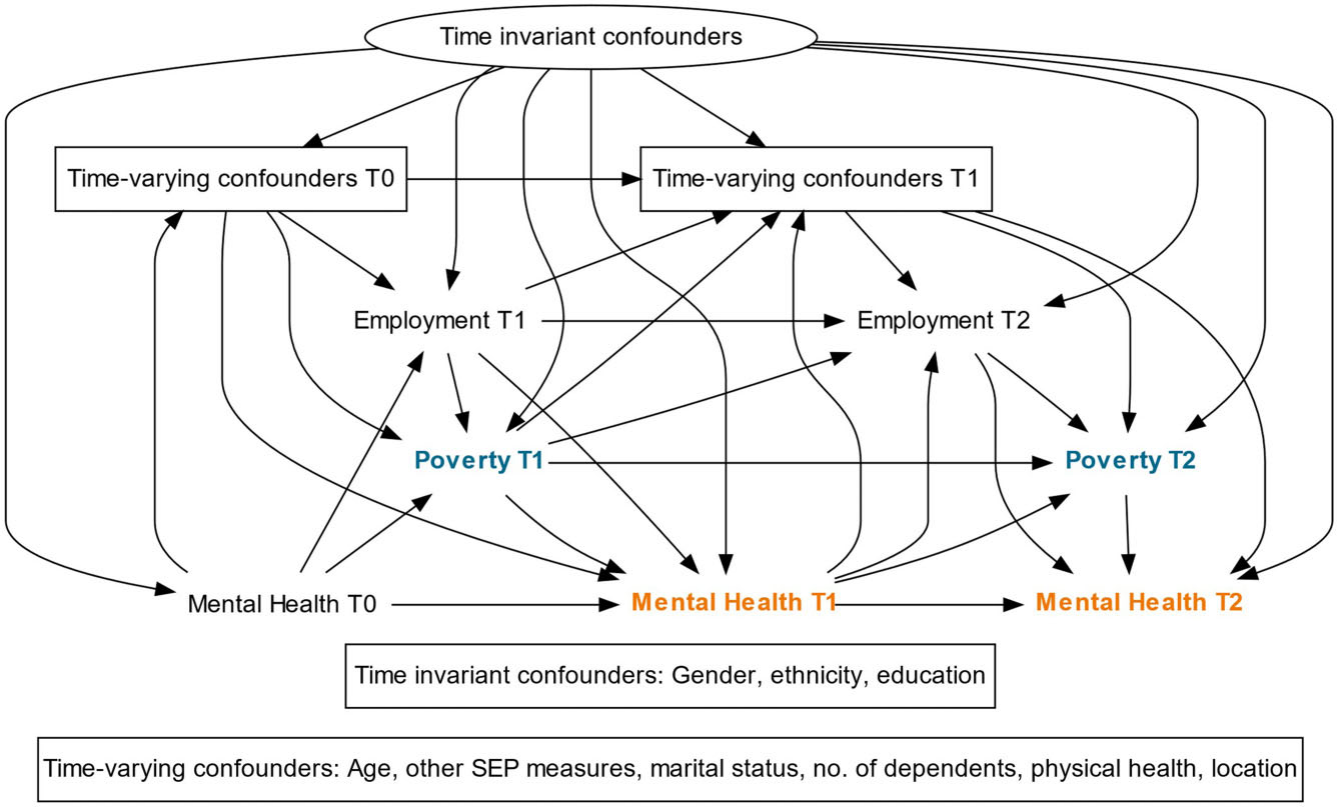
Directed acyclic graph (DAG) of the anticipated causal relationships between poverty (exposure), mental health (outcome), and time-invariant and time-varying confounders. T, time; SEP, socioeconomic position

We prepared a directed acyclic graph (DAG) based on existing literature to highlight our assumptions regarding the causal relationship between variables of interest, and to inform our statistical approach. In the DAG presented (Figure 1), each time point measures a sweep of data collection in the UKHLS; only three waves are shown for simplicity.

We examined the association between poverty and CMD within each sweep, after adjusting for both exposure and outcome in the previous sweep of data. We are therefore estimating the short-term impacts of poverty status on CMD, conditional only on the preceding year’s exposure status (rather than the effect of persistent poverty over time). We identified the minimally sufficient adjustment set for this short-term poverty effect at a time point (t) as being: all time-invariant confounders; all time-varying confounders at t-1; employment status at time t (see below); and mental health at t-1. We also adjusted for sweep number to ensure that we accounted for any time trend in the exposure.

### Time-invariant (baseline) confounders

Baseline confounders were self-reported gender (male/female), ethnicity (White/non-White), and highest educational attainment [high (degree-level), medium (General Certificate of Secondary Education, A-level or equivalent) or low (no formal qualifications)].

### Time-varying confounders

Several important time-varying confounders were identified a priori: paid employment (yes/no); housing tenure (owner/renter); receipt of welfare benefits (yes/no); marital status (single/coupled); number of children aged <16 years in the household; physical health [from the short-form survey (SF-12) physical component score (PCS)];^18^ and geography (government office region). Age was also considered a time-varying covariate, since the spacing between sweeps varied from household to household (included as a continuous variable and a squared term).

As shown in the DAG, time-varying confounders were included as lagged terms (i.e. at t-1), to ensure that they preceded the exposure, with one exception. Employment status was adjusted for at both time t and time t-1, since we hypothesized that changes in income resulting from employment would be more immediate than any effects of income on employment, and we also wished to account for employment trajectory from the previous to the current wave.

To ensure analyses adequately corrected for the influence of past exposure and outcome status (Figure 1), 1-year lagged versions of poverty status, GHQ caseness, and the mental health component of SF-12 were also included as time-varying confounders.

With all causal observational analyses there is potential for unmeasured or residual confounding to influence results, either from variables which are entirely unobserved or from variables which cannot be fully accounted for using the data available. In our case, potential sources of residual confounding would include historical disadvantage (e.g. adverse childhood experiences), changes in wealth or income security over time, and more nuanced measures of race/ethnicity.

### Effect modifiers

We considered three potentially important effect modifiers: gender, educational attainment, and age [dichotomized into younger (25–40 years) and older (41–64 years) working-age].

### Statistical analysis

To achieve our stated aim of determining the causal effect of poverty on mental health, the target causal parameter for our analysis was the causal risk difference: where Y is the outcome (CMD), and A is the exposure (poverty). Essentially, this represents the difference in the probability of CMD between those who were and were not experiencing poverty, if all else were equal. As a result, our required statistical estimand for analysis was: where W represents the set of measured confounding variables observed in our data. We used a double-robust approach, adjusting for baseline and time-varying confounding using inverse probability of treatment weights (IPTWs) and also adjusting for baseline and time-varying confounders in the outcome model.^19^ The double-robust approach means that if either the outcome or the weighting model is correctly specified, we will get an unbiased estimate.^20^ Under the assumption of no residual or unmeasured confounding, this estimate should represent the desired causal effect. We also estimated the causal odds ratio as a measure of the relative difference in effect size between the exposed and unexposed.


$$P\left(Y_{A=1}=1\right)\;–\;P\left(Y_{A=0}=1\right)$$

E{P(Y=1|A=1, W) – P(Y=1|A=0, W)}

We aimed to address the positivity assumption of causal inference (which states that there must be both exposed and unexposed individuals at every level of the confounders^21^) by interrogating the distribution of the confounders in the exposed and unexposed groups prior to calculation of the IPTWs, by limiting the number of categories within included variables where possible, and by stabilizing our IPTWs. To partially address the stable-unit-treatment-value-assumption or SUTVA (which requires both lack of interference of exposure between units and consistency^22^) we used measures of household income rather than individual income, as an individual’s income within a household will almost certainly impact on the exposure and outcome of others within the household. We appreciate that consistency may be a concern in our case, given that the experience of poverty is likely to differ between individuals and contexts, and similarly the effect of eradication of poverty on mental health may differ depending on precisely how poverty is eradicated. However, our causal estimates can hopefully be interpreted as an average of the effects of these different ‘types’ of poverty experiences, which we have identified using a commonly used definition of the exposure, and our stratified analyses consider some potential important differences between groups and could inform future research.

#### Multiple imputation

We imputed missing data between an individual’s first and last appearance in UKHLS, using multiple imputation with chained equations under a missing-at-random assumption.^23^ Observations with high missingness (those missing >9 of the 22 variables required for analysis, totalling 12.7% of all observations) were dropped. Due to the need for lagged data on time-varying confounders, the first wave of outcome data for each individual could not contribute to analysis, and these observations therefore only contributed confounder data. Twenty imputed datasets were then created with all variables including exposure, outcome, and lagged time-varying confounders.^24^

Gender, age, wave, and number of children were used as imputation variables; due to issues achieving model convergence, missing observations for region (*n* = 211, 0.0007%) were dropped. Income was logged prior to imputation to address non-normality, and interaction terms between income and the three effect modifiers were included in the imputation.^25^ Exposure and outcome variables were dichotomized after imputation. Supplementary Table S1 (available as Supplementary data at *IJE* online) details the regression models used to impute each included variable.

#### Marginal structural modelling

Inverse probability of treatment weights were calculated in an exposure model including all time invariant and time-varying confounders and a marker of the survey wave. To determine the adequacy of IPTWs, standardized mean differences (SMDs) between exposed and unexposed individuals were compared for all confounding variables before and after application of the weights, with residual SMD <0.2 reflecting reasonable balance and SMD <0.1 a negligible statistical difference.^26^ Applying these weights, we then calculated absolute risk differences in percentage points between exposed and unexposed groups and odds ratios using pooled logistic regression with clustered standard errors, with baseline and time-varying confounders included in the outcome model.^19^ We also calculated population attributable fractions (PAFs) to determine the percentage of CMD burden attributable to poverty status according to our causal estimate, using the standard equation^27^:


$$\mathrm{PAF}=\;\frac{\mathrm{Prevalence}\;in\;\mathrm{total}\;\mathrm{population}\;-\;\mathrm{Prevalence}\;in\;une\mathrm{xposed}}{\mathrm{Prevalence}\;in\;\mathrm{total}\;\mathrm{population}}$$

We ran additional models stratified by the three potential effect modifiers, recalculating IPTWs on the restricted sample following the approach outlined above. To test whether there was any differential effect magnitude for moves into poverty rather than out of poverty, we ran a separate analysis where the exposure variable was a transition into poverty since the previous wave of data collection, with the reference group restricted to include only those at risk of the outcome (i.e. not those already in poverty). The corresponding test for transitions out of poverty was also performed.

### Sensitivity analysis

For comparative purposes, we conducted a conditional fixed-effects logit regression with and without inclusion of the same lagged time-varying confounders, using post-estimation commands to generate the absolute difference in predicted probabilities. We also conducted a complete case analysis.

As we imputed missing data, our primary analyses did not incorporate the longitudinal survey weights for attrition/non-response provided by UKHLS (which restrict the sample to those present in every wave of data collection). However, as an additional sensitivity analysis we conducted the same analysis on this restricted sample with and without incorporation of the survey weights into our IPTWs.

Stata MP 16.1 was used for all analyses. Graphs were generated in R using ggplot2 and in Excel.

## Results

The final analytical sample consisted of 45 497 individuals across 202 297 observations; of these, 70.6% (*n* = 32 138; observations = 132 962) had complete data (see Supplementary Figure S1, available as Supplementary data at *IJE* online, for details on participant exclusion). The characteristics of the observed and imputed samples are displayed in Supplementary Table S2 (available as Supplementary data at *IJE* online). In comparison with complete cases, the imputed sample included slightly more males, non-White individuals, and those from lower socioeconomic backgrounds. Prevalence of both the exposure and outcome were higher in the imputed sample: for poverty 22.40% versus 19.52%; for presence of likely CMD 19.85% versus 18.94%.

### Balance of confounding variables

**Figure 2:**
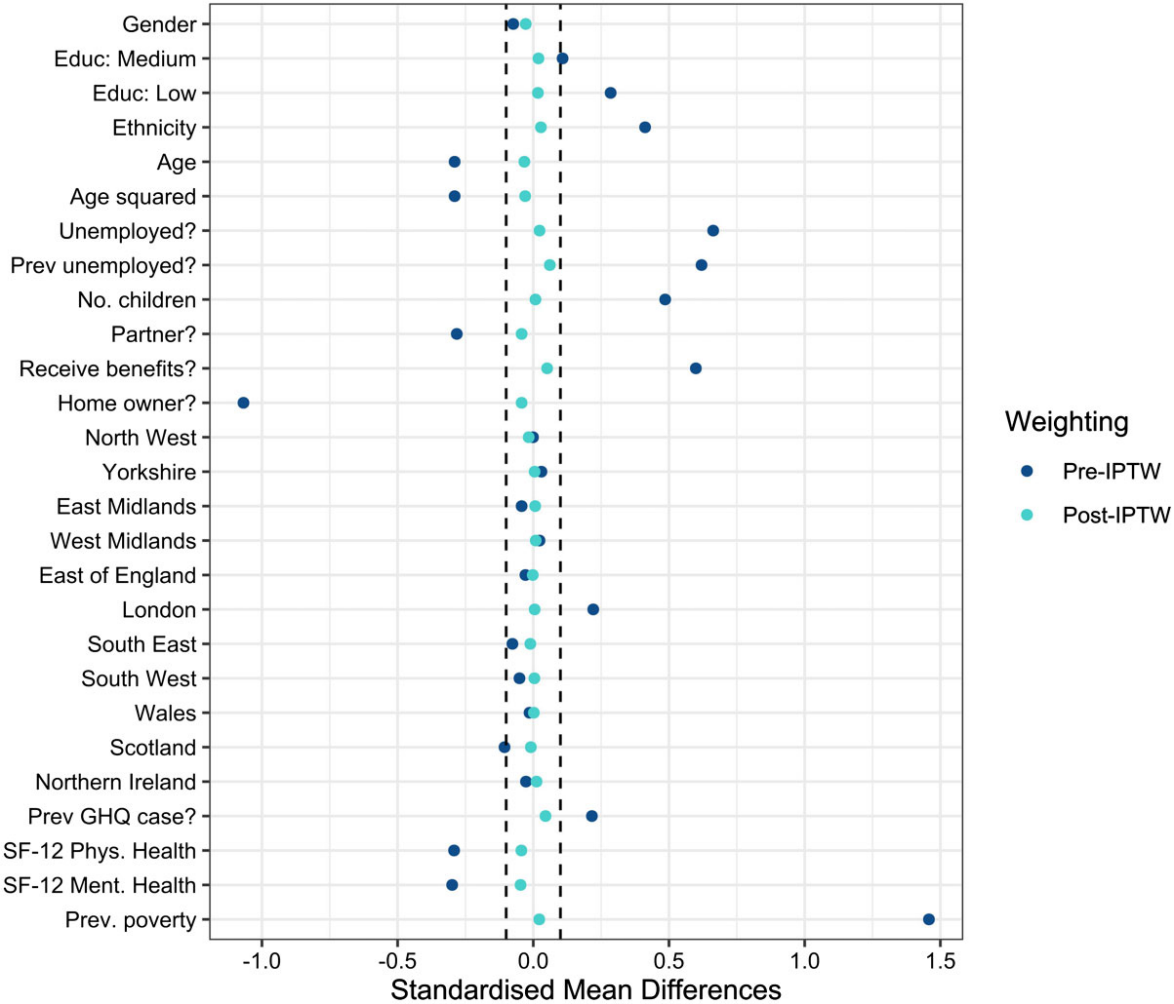
Balance of confounding variables before and after application of inverse probability of treatment weights (IPTWs) in primary analysis. Dotted lines indicate standardized mean differences (SMDs) between - 0.1 and 0.1, indicating a statistically negligible difference. SF-12, 12-item Short Form survey

Prior to weighting, all confounders other than gender and most regions were imbalanced between exposed and unexposed individuals (Figure 2; and Supplementary Table S3, available as Supplementary data at *IJE* online). Following application of IPTWs, good balance of confounders was achieved, with all SMDs ≤0.06 in the primary analysis. Good balance was also achieved in all stratified and sensitivity analyses, although for the analysis considering moves into poverty, the post-weighting SMD for previous employment status was slightly greater than what would be considered ‘negligible’, at 0.13 (Supplementary Table S3).

### Effect sizes for poverty

The absolute risk difference for poverty is estimated to be 2.15% [95% confidence interval (CI) 1.45, 2.84; %-point change] in the main analysis (Table 1), indicating that after balancing confounding factors using IPTWs, the prevalence of CMD is approximately 2% higher in those exposed to poverty than those who are not. Differentiating between transition type indicates that the detrimental impacts of moves into poverty [2.46% (95% CI 1.56, 3.36)] are larger than the benefits of moves out of poverty [–1.49% (95% CI –2.46, –0.53)]. Patterning is similar for the relative measure (using odds ratios). The population attributable fractions suggest that moves into poverty are responsible for 6.34% of the burden of CMD in the UK working-age population (95% CI 4.23, 8.45), whereas moves out of poverty reduce this burden by 2.81% (95% CI –4.46, –1.15).

**Table 1:** Causal effect estimates of poverty status on likelihood of common mental disorder (with 95% confidence intervals)

	Binary poverty status	Moving into poverty	Moving out of poverty
Odds ratio	1.17 (1.12, 1.24)	1.21 (1.13, 1.30)	0.90 (0.85, 0.97)
Absolute risk difference	2.15% (1.45%, 2.84%)	2.46% (1.56%, 3.36%)	−1.49% (–2.46%, –0.53%)
Prevalence in unexposed	20.59% (20.29%, 20.88%)	18.34% (18.12%, 18.55%)	27.29% (26.78%, 27.81%)
Population attr. fraction	4.78% (3.38%, 6.19%)	6.34% (4.23%, 8.45%)	−2.81% (–4.46%, –1.15%)
*n*	45 497 (202 297 obs)	39 772 (156 414 obs)	18 206 (45 769 obs)

Adjusted for both time invariant (gender, education, ethnicity) and time-varying confounders [age, age squared, employment status (current and 1 year lagged), benefit status (lagged), home ownership status (lagged), marital status (lagged), number of children (lagged), government office region (lagged), 12-item Short Form survey (SF-12) physical health component (lagged), SF-12 mental health component (lagged) and previous indication of common mental disorder (1 year lagged GHQ caseness)]. Absolute risk difference indicates %-point change. Columns 2 and 3 show results of sensitivity analysis separating transitions above and below the poverty threshold. Reference group for transition into poverty is those not in poverty in both years; reference group for transition out of poverty is those in poverty in both years.

Attr., attributable; obs, observations; GHQ, General Health Questionnaire.

### Stratification by potential effect modifiers

Poverty had a detrimental impact on CMD across all groups (Table 2). Absolute effects appeared larger in women than in men [2.34% (95% CI 1.41, 3.26) versus 1.73% (95% CI 0.72, 2.74)], though relative effects were similar. There was a slightly larger absolute effect for those with least education compared with those with high or medium education [2.20% (95% CI 0.90, 3.49) versus 2.13% (95% CI 0.85, 3.42) and 2.14% (95% CI 1.06, 3.22) respectively], though confidence intervals were wide. Both absolute and relative effects were similar for those of older and younger working age. Population attributable fractions were larger for those with high education [from 5.25% (95% CI 2.22, 8.28) versus 4.23% (95% CI 1.96, 6.51) for low education], though again confidence intervals were wide.

**Table 2:** Causal effect estimates of poverty status on likelihood of common mental disorder (with 95% confidence intervals), stratified by gender, highest education and age

	Sample size	Odds ratio	Absolute % diff.	Prev. in unexposed	PAF
Men	20 891 (90 369 obs)	1.16 (1.07, 1.26)	1.73% (0.72, 2.74)	17.15% (16.75, 17.54)	4.64% (2.23, 7.05)
Women	24 634 (111 928 obs)	1.17 (1.10, 1.25)	2.34% (1.41, 3.26)	23.25% (22.85, 23.65)	4.62% (2.87, 6.36)
High education	19 987 (82 080 obs)	1.18 (1.07, 1.30)	2.13% (0.85, 3.42)	18.53% (18.16, 18.91)	5.25% (2.22, 8.28)
Medium education	19 576 (75 559 obs)	1.18 (1.09, 1.28)	2.14% (1.06, 3.22)	20.39% (19.91, 20.86)	4.84% (2.70, 6.99)
Low education	12 636 (44 658 obs)	1.17 (1.07, 1.28)	2.20% (0.90, 3.49)	24.11% (23.38, 24.83)	4.23% (1.96, 6.51)
Younger working-age	20 384 (71 777 obs)	1.16 (1.07, 1.25)	2.05% (0.96, 3.14)	20.52% (19.97, 21.08)	4.59% (2.27, 6.91)
Older working-age	29 711 (130 520 obs)	1.18 (1.10, 1.26)	2.14% (1.25, 3.03)	20.68% (20.34, 21.01)	4.75% (2.94, 6.56)

Adjusted for both tim- invariant (gender, education, ethnicity) and time-varying confounders [age, age squared, employment status (current and 1 year lagged), benefit status (lagged), home ownership status (lagged), marital status (lagged), number of children (lagged), government office region (lagged), 12-item Short Form survey (SF-12) physical health component (lagged), SF-12 mental health component (lagged), and previous indication of common mental disorder (1 year lagged GHQ-12 caseness)]. Absolute risk difference indicates %-point change.

Obs, observations; Prev., prevalence; diff., difference; PAF, population attributable fraction; GHQ-12, General Health Questionnaire.

**Figure 3:**
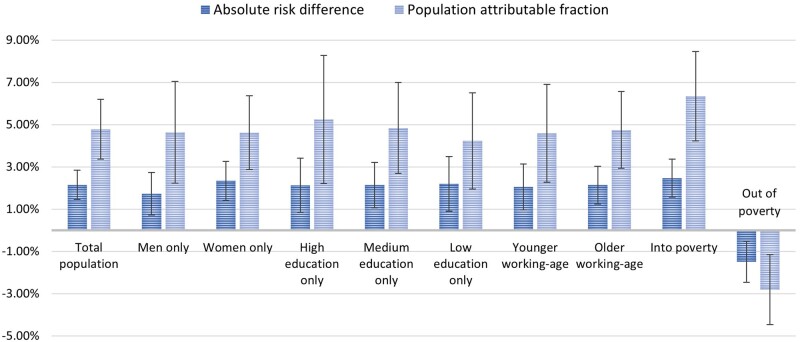
Absolute risk difference and population attributable fractions for all causal estimates of the relationship between poverty status and likelihood of common mental disorder, with 95% confidence intervals

Figure 3 illustrates the absolute risk differences and PAFs across all main analyses with 95% CIs, demonstrating the larger effects in women than men for both measures, for those with least education, and for transitions into rather than out of poverty.

### Sensitivity analyses

In our comparative analyses (Supplementary Table S4, available as Supplementary data at *IJE* online), the MSM effect size was close to the adjusted fixed-effects estimates in most analyses, though occasionally it fell between this and the unadjusted fixed-effects estimates. The confidence intervals around the MSM estimates were more precise than those generated by the fixed-effects models. Effect sizes were higher in complete case analyses compared with results from the imputed sample for all modelling approaches (Supplementary Table S5, available as Supplementary data at *IJE* online), but the differences for the MSM analysis were particularly small compared with other estimation methods [2.29% (95% CI 1.45, 3.12) in complete cases versus 2.15% (95% CI 1.45, 2.84) in the imputation sample]. In the restricted sample of UKHLS who participated in all nine survey waves and have longitudinal weights provided (Supplementary Table S6, available as Supplementary data at *IJE* online), MSM results were again fairly similar to those from the complete case and imputed sample analyses [2.52% (95% CI 1.40, 3.64) without incorporating these weights into IPTWs and 2.61% (95% CI 1.35, 3.88) incorporating them]. Estimates from fixed-effect models were less consistent across the different samples (Supplementary Tables S5 and S6).

## Discussion

Using causally informed methods, we find that the average treatment effect of poverty status on likelihood of experiencing a common mental disorder in the UK population is 2.15% (%-point change; 95% CI 1.45, 2.84), and that the negative effect of moving below the poverty line is larger than the mental health improvement from moving out of poverty [2.46% (95% CI 1.56, 3.36) versus -1.49% (95% CI -2.46, -0.53)]. Interpreting our estimates causally suggests that moves into poverty cause 6.34% of the current burden of poor mental health in the UK population (95% CI 4.23, 8.45). Larger effects were seen for women than men, and possibly for those with lower educational attainment. In our sensitivity analyses, double-robust marginal structural modelling seemed to provide more consistent estimates across different samples compared with fixed-effects regression, with more precision.

Our finding of an average treatment effect for poverty of around 2%, with an odds ratio of 1.2, is in keeping with existing literature on income and mental health. Our recent systematic review and meta-analysis found that income changes which moved individuals across a poverty line had an average effect of SMD 0.13 (equivalent to an odds ratio of 1.27).^5^ Our estimate also aligns well with a review by Ridley *et al*. which focused specifically on randomized trials of anti-poverty interventions and reported a meta-analysed effect size of SMD 0.09 (odds ratio 1.18),^28^^,^^29^ suggesting that our methodological approach has, we hope, been successful in reducing the considerable bias inherent in studying this exposure-outcome combination in our observational data.

The presence of an asymmetrical relationship where ‘[income] losses loom harder than gains’ when considering one’s future wellbeing is a well-established phenomenon within economics, known as loss aversion.^30^ It has been demonstrated empirically that this is not only speculative, but also translates to the subsequent experienced losses having a greater magnitude of effect on general wellbeing than the equivalent income gains.^31^ For mental health more specifically, our review of the relationship between income changes and mental health outcomes also reported larger effect sizes for income losses than income increases.^5^ Though there was low certainty in this finding, our replication of it here using causal methods potentially adds weight to the hypothesis, which would have clear implications for policy making.

The finding of a larger effect of poverty on women than men is in keeping with findings in some other contexts/settings,^32^^,^^33^ though there remains a paucity of research examining this.^4^ The slightly larger effect in those with least education is not unexpected, as prior literature had suggested larger effects in the most deprived, though we note the wide confidence intervals around these estimates preclude the drawing of definitive conclusions.^5^^,^^34^

There is considerable literature on the relative benefits and disadvantages of different philosophical and statistical approaches to estimating causal effects.^35–38^ Whereas fixed-effects models are often noted to be particularly useful in addressing the issue of unmeasured baseline confounding,^39^ they are not well placed to account for intermediate confounding, carrying the same assumptions as traditional multivariable regression modelling.^40^ In a situation such as ours where these assumptions are clearly not met (Figure 1) and important baseline confounders are measured, marginal structural models may result in less biased estimates.^12^ In line with these criticisms, findings from our sensitivity analyses suggested that in some circumstances fixed-effects models either under-or over-estimated our MSM-derived causal effect, depending on whether adjustment is made for intermediate confounders. A recent review considering the effect of income on children’s health reported smaller effects in studies using fixed-effects methods in comparison with those using experimental or quasi-experimental methods,^41^ adding further weight to the argument that they may perform less well in these circumstances.

Finally, the cost of poor mental health to UK employers is estimated to be between £42bn and £45bn per year in early 2020,^42^ with the NHS in England spending £14bn per year (14.8% of its local health spend) on mental health provision for those with diagnosed mental health problems.^43^ If ∼6% of this spend is directly due to the effect of poverty on working people’s mental health, the theoretical savings associated with removal of this exposure from the population would be in the region of £3.6bn/year, though caution should be taken in this interpretation as our outcome measure is a marker of likely rather than diagnosed mental health problems.

### Strengths and limitations

Our study has several important strengths. We used nine waves of longitudinal data from a UK-representative cohort to generate a large sample followed over a decade, and used multiple imputation to reduce the impact of attrition and item missingness on our findings. We pre-specified key confounders in a DAG to make clear our assumptions regarding the causal relationships of interest. We took a causally informed approach to statistical analysis based on this DAG, and included confounders in both exposure and outcome regression models (known as ‘double-robust’). We also report multiple sensitivity analyses to consider bias in our chosen methods and provide comparisons with more traditional approaches to analysis.

However, our study does have some limitations. Causal interpretation of our estimates requires an assumption of no unmeasured or residual confounding, and although we have included indicators for all proposed confounders as far as the data allow, there may be some which are not fully represented by our set of measured variables and others which we had not identified. The presence of unmeasured or residual confounding such as this could lead to bias of unclear direction, which could substantially affect the results. Confidence intervals are wide in our stratified models, reducing the ability to draw definitive conclusions on effect modification. The use of a binary exposure assumes that any experience of poverty exerts a similar effect, which is likely to be an over-simplification of the nuanced experiences of those living with different levels and contexts of income poverty. We also note that we have chosen to focus on the short-term or instantaneous effects of poverty on mental health; this is certainly of policy interest, but it does not allow us to incorporate the complexity of prolonged exposure to poverty over time, or any effect of repeated or historical poverty exposures, which would be of interest to explore in future research. We also elected to include only employment status as a confounding variable in the concurrent sweep of data collection (due to the immediacy of its effect on poverty status) rather than any other time-varying confounders such as marital status, as we felt their inclusion risked over-adjustment or conditioning on a mediator; however, we appreciate that this is an assumption, and that different approaches could be taken. Due to small numbers, ethnicity, housing tenure, and marital status were dichotomized during imputation, introducing measurement error and, potentially, residual confounding. This is a particular concern for the ethnicity variable, where some nuance is likely to have been eliminated by collapsing the categories. We also required the exclusion of observations with large amounts of missing data to achieve model convergence during imputation, though our sensitivity analysis suggests this did not affect the representativeness of the imputed sample.

### Policy implications

Our findings add to the evidence base suggesting that poverty does have an effect on mental health, overcoming methodological criticisms posed by those who have argued that no effect exists. In fact, our PAFs suggest that if poverty were eradicated, the prevalence of common mental health problems in the UK population would be 6.3% lower. This suggests that policy makers should design income and welfare policies that protect working-age individuals from falling into poverty, especially given that any deleterious effects of this on mental health may not be entirely reversed by lifting someone back out of poverty in the future. Particularly close policy attention may be needed for women, and potentially those with least education. In addition, these causal estimates may be of practical use in policy or economic modelling to more accurately predict the impact of planned policy changes on mental health.

### Areas for future research

Replication of our methods in other populations would be useful to determine the degree to which causal relationships between income and mental health may differ between settings and contexts, and to explore the effects of longer-term exposure to poverty. Given the consistency of our findings across samples in comparison with traditional regression, we believe our methods could also be usefully applied and extended to consider other social determinants of mental health with similarly complex causal structures.

## Conclusions

Our analysis suggests poverty is currently responsible for around ∼6% of the burden of poor mental health in the UK working-age population, at considerable economic cost. Policy makers must consider the economic and health consequences of not protecting adults from falling below the poverty line, particularly women and those with least education.

## Ethics approval

The University of Essex Ethics Committee has approved all data collection on the Understanding Society main study and innovation panel waves, including asking consent for all data linkages except to health records. Requesting consent for health record linkage was approved at Wave 1 by the National Research Ethics Service (NRES) Oxfordshire REC A (08/H0604/124), at BHPS Wave 18 by the NRES Royal Free Hospital & Medical School (08/H0720/60) and at Wave 4 by NRES Southampton REC A (11/SC/0274). Approval for the collection of biosocial data by trained nurses in Waves 2 and 3 of the main survey was obtained from the National Research Ethics Service (Understanding Society—UK Household Longitudinal Study: A Biosocial Component, Oxfordshire A REC, Reference: 10/H0604/2). No further approval was required for the current analysis of the existing data.

## Supplementary Material

dyac226_Supplementary_Data

## Data Availability

Original UKHLS data are held by the UK Data Service and are available on request from (http://doi.org/10.5255/UKDA-SN-6614-14). The analytical codes are available in an online repository from (https://github.com/rachelmthomson/thomson-msm-mh).
