# Supplementary material for: Effects of poverty on mental health in the UK working-age population: causal analyses of the UK Household Longitudinal Study
Source: Int J Epidemiol. 2022 Dec 8;52(2):512–22. doi: 10.1093/ije/dyac226 (PMC10114108; doi:10.1093/ije/dyac226)

Supplementary Material

Table S1: Variables included within the imputation model

REGRESSION MODEL	VARIABLES	% MISSINGNESS
LINEAR	Log(household income)	2.60%
	One-year lagged log(household income)	2.67%
	Interaction: log(income) x gender	2.60%
	Interaction: log(income) x education	13.98%
	Interaction: log(income) x age	2.60%
TRUNCATED LINEAR (RANGE 0 TO 100)	SF-12 physical component score (PCS)	12.41%
	One-year lagged SF-12 PCS	12.73%
	SF-12 mental component score (MCS)	12.41%
	One-year lagged SF-12 MCS	12.73%
LOGISTIC	Employment status	5.21%
	One-year lagged employment status	4.67%
	If in receipt of benefits (benefit status)	0.48%
	One-year lagged benefit status	0.34%
	Home ownership status	1.11%
	One-year lagged home ownership status	0.97%
	Marital status (coupled versus not coupled)	0.13%
	One-year lagged marital status	0.13%
	Ethnicity (white/non-white)	1.75%
ORDERED LOGIT	GHQ-12 score	11.66%
	One-year lagged GHQ-12 score	12.76%
	Highest education	11.67%
COMPLETE (IMPUTATION VARIABLES)	Gender	0%
	Government office region	0%
	One-year lagged government office region	0%
	Number of own children in household	0%
	One-year lagged number of children	0%
	Study wave	0%

Table S2: Characteristics of included observations

	**COMPLETE CASES** n = 32,138; obs = 132,962		**IMPUTED DATA** n = 45,497; obs = 202,297	
	Continuous variables: Mean (SD), Range			
Age	45.36 (10.90)	25.00 to 64.00	45.31 (10.93)	25.00 to 64.00
Monthly household income	£1633.34 (1707.72)	£0.04 to £201671	£1568.82 (2015.52)	£0.04 to £202620
Household income (t-1)	£1581.62 (1773.26)	£0.04 to £202620	£1519.30 (2019.67)	£0.04 to £220830
GHQ-12 score	1.81 (3.08)	0.00 to 12.00	1.88 (3.13)	0.00 to 12.00
GHQ-12 score (t-1)	1.81 (3.05)	0.00 to 12.00	1.89 (3.11)	0.00 to 12.00
Number of children	0.71 (1.01)	0.00 to 9.00	0.73 (1.05)	0.00 to 9.00
Number of children (t-1)	0.72 (1.02)	0.00 to 9.00	0.74 (1.05)	0.00 to 9.00
SF-12 physical health	51.09 (10.14)	4.64 to 76.29	50.61 (10.45)	2.34 to 90.76
SF-12 physical health (t-1)	51.31 (10.00)	4.48 to 76.29	50.85 (10.32)	3.81 to 91.26
SF-12 mental health	48.80 (9.87)	0.00 to 76.62	48.58 (10.04)	0.00 to 80.81
SF-12 mental health (t-1)	49.07 (9.80)	0.00 to 77.09	48.85 (9.98)	0.00 to 81.89
	Categorical variables: Frequency (%)			

Gender	Male	57,051 (42.91%)	Male	90,369 (44.67%)
	Female	75,911 (57.09%)	Female	111,928 (55.33%)
Educational attainment	High	56,924 (42.81%)	High	82,080 (40.57%)
	Medium	49,886 (37.52%)	Medium	75,559 (37.35%)
	Low	26,152 (19.67%)	Low	44,658 (22.08%)
Age group	Younger (25-40)	47,023 (35.37%)	Younger (25-40)	71,777 (35.48%)
	Older (41-64)	85,939 (64.63%)	Older 41-64)	130,520 (64.52%)
Ethnicity	White	114,778 (86.32%)	White	166,621 (82.36%)
	Non-white	18,184 (13.68%)	Non-white	35,676 (17.64%)
In poverty?	Yes	25,954 (19.52%)	Yes	45,305 (22.40%)
	No	107,008 (80.48%)	No	156,992 (77.60%)
In poverty? (t-1)	Yes	26,231 (19.73%)	Yes	45,830 (22.65%)
	No	106,731 (80.27%)	No	156,467 (77.35%)
Poverty transition	Never in poverty	97,690 (73.47%)	Never in poverty	141,576 (69.98%)
	Moved out of poverty	9,318 (7.01%)	Moved out of poverty	15,417 (7.62%)
	Moved into poverty	9,041 (6.80%)	Moved into poverty	14,891 (7.36%)
	Persistent poverty	16,913 (12.72%)	Persistent poverty	30,413 (15.03%)
GHQ-12 caseness	Yes	25,185 (18.94%)	Yes	40,164 (19.85%)
	No	107,777 (81.06%)	No	162,133 (80.15%)
GHQ-12 caseness (t-1)	Yes	25,102 (18.88%)	Yes	40,177 (19.86%)
	No	107,860 (81.12%)	No	162,120 (80.14%)
In employment?	Yes	103,700 (77.99%)	Yes	150,418 (74.35%)
	No	29,262 (22.01%)	No	51,879 (25.65%)
In employment? (t-1)	Yes	104,115 (78.30%)	Yes	151,007 (74.65%)
	No	28,847 (21.70%)	No	51,290 (25.35%)
Receives benefits?	Yes	83,508 (62.81%)	Yes	131,899 (65.20%)
	No	49,454 (37.19%)	No	70,398 (34.80%)
Receives benefits? (t-1)	Yes	83,335 (62.68%)	Yes	131,560 (65.03%)
	No	49,627 (37.32%)	No	70,737 (34.97%)
Owns home?	Yes	98,228 (73.88%)	Yes	143,339 (70.86%)
	No	34,734 (26.12%)	No	58,958 (29.14%)
Owns home? (t-1)	Yes	97,760 (73.52%)	Yes	142,772 (70.58%)
	No	35,202 (26.48%)	No	59,525 (29.42%)
Has partner?	Yes	99,408 (74.76%)	Yes	149,911 (74.10%)
	No	33,554 (25.24%)	No	52,387 (25.90%)
Has partner? (t-1)	Yes	99,027 (74.48%)	Yes	149,408 (73.86%)
	No	33,935 (25.52%)	No	52,889 (26.14%)
Govt. office region	North East	5,548 (4.17%)	North East	7,596 (3.75%)
	North West	14,495 (10.90%)	North West	20,490 (10.13%)
	Yorkshire & the Humber	11,152 (8.39%)	Yorkshire & the Humber	16,681 (8.25%)
	East Midlands	10,398 (7.82%)	East Midlands	15,057 (7.44%)
	West Midlands	11,359 (8.54%)	West Midlands	16,538 (8.18%)
	East of England	12,281 (9.24%)	East of England	17,214 (8.51%)
	London	14,326 (10.77%)	London	26,806 (13.25%)
	South East	17,150 (12.90%)	South East	23,884 (11.81%)
	South West	12,041 (9.06%)	South West	15,917 (7.87%)
	Wales	7,507 (5.65%)	Wales	12,887 (6.37%)
	Scotland	10,833 (8.15%)	Scotland	16,846 (8.33%)
	Northern Ireland	5,872 (4.42%)	Northern Ireland	12,381 (6.12%)

Govt. office region (t-1)	North East	5,541 (4.17%)	North East	7,590 (3.75%)
	North West	14,500 (10.91%)	North West	20,488 (10.13%)
	Yorkshire & the Humber	11,142 (8.38%)	Yorkshire & the Humber	16,669 (8.24%)
	East Midlands	10,380 (7.81%)	East Midlands	15,042 (7.44%)
	West Midlands	11,334 (8.52%)	West Midlands	16,523 (8.17%)
	East of England	12,254 (9.22%)	East of England	17,163 (8.48%)
	London	14,432 (10.85%)	London	26,999 (13.35%)
	South East	17,174 (12.92%)	South East	23,860 (11.79%)
	South West	12,001 (9.03%)	South West	15,870 (7.84%)
	Wales	7,513 (5.65%)	Wales	12,887 (6.37%)
	Scotland	10,820 (8.14%)	Scotland	16,822 (8.32%)
	Northern Ireland	5,871 (4.42%)	Northern Ireland	12,384 (6.12%)

Table S3: Standardised mean differences of confounding variables between exposed and unexposed groups in primary and stratified analyses, before and after application of inverse probability of treatment weights (IPTWs)

	Whole imputed sample		Complete cases		Into poverty		Out of poverty		Men		Women	
	Pre-IPTWs	Post-IPTWs	Pre-IPTWs	Post-IPTWs	Pre-IPTWs	Post-IPTWs	Pre-IPTWs	Post-IPTWs	Pre-IPTWs	Post-IPTWs	Pre-IPTWs	Post-IPTWs
Gender	-0.074	-0.028	-0.079	-0.035	-0.055	-0.031	0.004	-0.007	.	.	.	.
Educ: Medium	0.108	0.019	0.167	0.027	0.096	0.016	-0.027	0.000	0.060	0.023	0.147	0.018
Educ: Low	0.285	0.017	0.244	0.010	0.182	0.026	-0.087	0.000	0.283	0.008	0.290	0.024
Ethnicity	0.412	0.028	0.293	0.031	0.288	0.040	-0.096	0.004	0.412	0.030	0.410	0.027
Age	-0.290	-0.033	-0.294	-0.040	-0.212	-0.016	0.098	0.005	-0.200	-0.038	-0.356	-0.028
Age squared	-0.290	-0.030	-0.291	-0.036	-0.208	-0.014	0.101	0.005	-0.200	-0.037	-0.356	-0.025
Unemployed?	0.663	0.023	0.653	0.013	0.475	0.036	-0.296	-0.003	0.602	0.010	0.700	0.029
Prev unemployed?	0.620	0.061	0.601	0.065	0.289	0.125	-0.078	-0.011	0.537	0.069	0.672	0.055
No. children	0.486	0.008	0.471	0.013	0.302	0.025	-0.194	0.000	0.428	0.004	0.526	0.015
Partner?	-0.282	-0.043	-0.319	-0.039	-0.190	-0.058	0.043	0.003	-0.148	-0.051	-0.371	-0.041
Receive benefits?	0.599	0.051	0.631	0.049	0.412	0.038	-0.149	0.000	0.587	0.039	0.603	0.057
Homeowner?	-1.068	-0.043	-1.059	-0.045	-0.652	-0.035	0.274	0.005	-1.024	-0.042	-1.099	-0.045
North West	-0.001	-0.017	0.010	-0.019	-0.007	-0.020	-0.005	0.001	0.003	-0.022	-0.004	-0.014
Yorkshire	0.030	0.005	0.033	0.009	0.022	0.003	0.001	-0.002	0.031	0.007	0.031	0.005
East Midlands	-0.043	0.007	-0.033	0.012	-0.029	0.006	0.022	-0.001	-0.031	0.012	-0.051	0.005
West Midlands	0.023	0.009	0.022	0.011	0.010	0.010	-0.012	-0.001	0.014	0.010	0.030	0.008
East of England	-0.029	-0.002	-0.014	-0.007	-0.012	-0.008	0.004	-0.004	-0.027	0.004	-0.030	-0.006
London	0.221	0.005	0.114	0.002	0.145	0.011	-0.060	0.000	0.206	0.003	0.231	0.008
South East	-0.077	-0.011	-0.057	-0.012	-0.053	-0.007	0.010	0.002	-0.059	-0.016	-0.091	-0.009
South West	-0.051	0.004	-0.021	0.015	-0.024	0.003	0.034	0.002	-0.056	0.011	-0.046	-0.001
Wales	-0.014	0.002	0.007	0.006	-0.012	0.009	-0.001	-0.001	-0.012	0.001	-0.015	0.002
Scotland	-0.106	-0.009	-0.075	-0.009	-0.060	-0.017	0.036	0.002	-0.106	-0.009	-0.106	-0.008
Northern Ireland	-0.027	0.012	-0.024	0.005	-0.011	0.019	0.003	0.003	-0.027	0.014	-0.028	0.011
Prev GHQ case?	0.216	0.045	0.226	0.052	0.146	0.058	-0.016	0.000	0.217	0.040	0.207	0.045
SF-12 Phys. Health	-0.292	-0.044	-0.291	-0.046	-0.227	-0.053	0.037	-0.009	-0.325	-0.021	-0.265	-0.058
SF-12 Ment. Health	-0.299	-0.047	-0.319	-0.050	-0.208	-0.064	0.052	-0.006	-0.289	-0.048	-0.297	-0.042
Prev. poverty	1.458	0.022	1.442	0.022	.	.	.	.	1.467	0.015	1.447	0.029

	High education		Medium education		Low education		Younger working-age		Older working-age	
	Pre-IPTWs	Post-IPTWs	Pre-IPTWs	Post-IPTWs	Pre-IPTWs	Post-IPTWs	Pre-IPTWs	Post-IPTWs	Pre-IPTWs	Post-IPTWs
Gender	-0.034	-0.038	-0.130	-0.021	-0.092	-0.025	-0.152	-0.023	-0.009	-0.032
Educ: Medium	.	.	.	.	.	.	0.221	0.018	0.027	0.024
Educ: Low	.	.	.	.	.	.	0.289	-0.010	0.344	0.036
Ethnicity	0.541	0.045	0.427	0.021	0.382	0.010	0.348	0.002	0.425	0.041
Age	-0.326	-0.064	-0.427	-0.034	-0.318	0.007	-0.035	-0.036	-0.186	-0.007
Age squared	-0.334	-0.061	-0.427	-0.032	-0.317	0.008	-0.035	-0.036	-0.182	-0.006
Unemployed?	0.529	0.011	0.611	0.016	0.687	0.033	0.774	0.010	0.648	0.035
Prev unemployed?	0.468	0.081	0.572	0.045	0.654	0.054	0.707	0.042	0.601	0.078
No. children	0.495	0.023	0.564	0.016	0.459	-0.023	0.621	-0.006	0.299	0.014
Partner?	-0.244	-0.062	-0.246	-0.037	-0.333	-0.021	-0.087	-0.014	-0.405	-0.052
Receive benefits?	0.509	0.047	0.600	0.044	0.555	0.047	0.616	0.034	0.569	0.065
Homeowner?	-0.984	-0.052	-1.051	-0.034	-1.048	-0.040	-0.994	-0.031	-1.059	-0.047
North West	-0.002	-0.019	-0.012	-0.024	0.021	-0.005	-0.004	-0.006	-0.007	-0.025
Yorkshire	0.009	0.013	0.007	0.005	0.049	0.001	0.015	0.010	0.034	0.002
East Midlands	-0.066	-0.011	-0.051	0.023	-0.052	0.000	-0.029	-0.021	-0.052	0.022
West Midlands	-0.013	0.011	0.006	0.006	0.065	0.015	0.015	0.007	0.027	0.008
East of England	-0.003	-0.016	-0.033	0.005	-0.034	-0.003	-0.006	-0.006	-0.047	0.000
London	0.298	0.015	0.251	-0.003	0.263	0.008	0.175	-0.013	0.239	0.016
South East	-0.076	-0.022	-0.037	-0.003	-0.053	0.004	-0.058	0.004	-0.087	-0.017
South West	-0.047	0.016	-0.051	0.001	-0.030	-0.011	-0.022	0.028	-0.060	-0.010
Wales	-0.054	0.006	-0.003	0.002	-0.077	-0.003	-0.008	-0.005	-0.015	0.007
Scotland	-0.126	-0.014	-0.087	-0.001	-0.168	-0.022	-0.103	0.002	-0.096	-0.013
Northern Ireland	-0.082	0.026	-0.058	0.006	-0.031	0.011	-0.053	0.008	-0.003	0.016
Prev GHQ case?	0.166	0.058	0.221	0.039	0.217	0.030	0.162	0.019	0.259	0.061
SF-12 Phys. Health	-0.253	-0.030	-0.213	-0.039	-0.230	-0.040	-0.299	-0.020	-0.382	-0.067
SF-12 Ment. Health	-0.232	-0.062	-0.310	-0.028	-0.305	-0.045	-0.210	-0.019	-0.344	-0.062
Prev. poverty	1.374	0.026	1.427	0.018	1.443	0.017	1.438	0.009	1.443	0.027

Highlighted cells are those with standardised mean difference ≥ 0.1, indicating a greater than negligible statistical difference between exposed and unexposed groups

Table S4: Comparative analysis contrasting use of marginal structural modelling (MSM) versus fixed-effects (FE) modelling to estimate causal effects of poverty status on likelihood of common mental disorder

	Sample size	FE sample size	Measure	Unadj. estimate	FE estimate	FE estimate, adj.	MSM estimate
**Full sample**	45,497	16,207	odds ratio	1.73 (1.67, 1.79)	1.22 (1.16, 1.29)	1.16 (1.10, 1.23)	1.17 (1.12, 1.24)
	(202,297 obs)	(94,481 obs)	% change	9.45% (8.83%, 10.08%)	5.02% (3.75%, 6.28%)	1.49% (0.25%, 2.72%)	2.15% (1.45%, 2.84%)
**Complete cases**	32,138	10,146	odds ratio	1.80 (1.73, 1.88)	1.29 (1.21, 1.37)	1.23 (1.15, 1.31)	1.19 (1.12, 1.27)
	(132,962 obs)	(56,070 obs)	% change	9.97% (9.20%, 10.73%)	6.28% (4.68%, 7.87%)	4.09% (1.73%, 6.45%)	2.29% (1.45%, 3.12%)
**Into poverty**	39,772	12,183	odds ratio	1.65 (1.58, 1.73)	1.25 (1.17, 1.35)	1.20 (1.11, 1.29)	1.21 (1.13, 1.30)
	(156,414 obs)	(65,685 obs)	% change	8.35% (7.55%, 9.14%)	5.64% (3.87%, 7.41%)	2.17% (0.11%, 4.23%)	2.46% (1.56%, 3.36%)
**Out of poverty**	18,206	4,107	odds ratio	0.79 (0.75, 0.83)	0.83 (0.76, 0.91)	0.88 (0.80, 0.97)	0.90 (0.85, 0.97)
	(45,769 obs)	(17,247 obs)	% change	-4.58% (-5.60%, -3.55%)	-4.51% (-6.70%, -2.32%)	-2.29% (-5.11%, 0.52%)	-1.49% (-2.46%, -0.53%)
**Men**	20,891	6,545	odds ratio	1.80 (1.71, 1.90)	1.27 (1.17, 1.39)	1.17 (1.07, 1.28)	1.16 (1.07, 1.26)
	(90,369 obs)	(37,354 obs)	% change	8.95% (8.04%, 9.86%)	6.01% (3.94%, 8.09%)	0.49% (-0.38%, 1.36%)	1.73% (0.72%, 2.74%)
**Women**	24,634	9,656	odds ratio	1.66 (1.59, 1.73)	1.19 (1.12, 1.27)	1.16 (1.08, 1.24)	1.17 (1.10, 1.25)
	(111,928 obs)	(57,289 obs)	% change	9.46% (8.61%, 10.30%)	4.39% (2.77%, 6.01%)	2.36% (0.45%, 4.27%)	2.34% (1.41%, 3.26%)
**High education**	19,987	6,409	odds ratio	1.57 (1.48, 1.67)	1.28 (1.17, 1.41)	1.23 (1.11, 1.35)	1.18 (1.07, 1.30)
	(82,080 obs)	(36,828 obs)	% change	7.32% (6.25%, 8.40%)	6.23% (3.96%, 8.50%)	1.78% (-0.63%, 4.19%)	2.13% (0.85%, 3.42%)
**Medium education**	19,576	5,940	odds ratio	1.76 (1.67, 1.86)	1.20 (1.10, 1.31)	1.15 (1.05, 1.26)	1.18 (1.09, 1.28)
	(75,559 obs)	(32,754 obs)	% change	9.70% (8.73%, 10.68%)	4.55% (2.41%, 6.68%)	2.56% (-0.06%, 5.18%)	2.14% (1.06%, 3.22%)
**Low education**	12,636	3,842	odds ratio	1.68 (1.58, 1.79)	1.22 (1.10, 1.36)	1.16 (1.04, 1.29)	1.17 (1.07, 1.28)
	(44,658 obs)	(19,980 obs)	% change	9.63% (8.42%, 10.85%)	5.02% (2.44%, 7.61%)	2.21% (-1.02%, 5.44%)	2.20% (0.90%, 3.49%)
**Younger working-age (25-40 years)**	20,384	6,285	odds ratio	1.53 (1.45, 1.61)	1.18 (1.09, 1.29)	1.12 (1.03, 1.22)	1.16 (1.07, 1.25)
	(71,777 obs)	(31,655 obs)	% change	7.14% (6.25%, 8.03%)	4.20% (2.07%, 6.32%)	0.20% (-0.37%, 0.76%)	2.05% (0.96%, 3.14%)
**Older working-age (41-64 years)**	29,711	10,168	odds ratio	1.90 (1.82, 1.98)	1.26 (1.18, 1.36)	1.21 (1.12, 1.29)	1.18 (1.10, 1.26)
	(130,520 obs)	(58,737 obs)	% change	11.25% (10.40%, 12.10%)	5.84% (4.12%, 7.56%)	3.48% (0.09%, 6.87%)	2.14% (1.25%, 3.03%)

FE sample size excludes those with no variation in outcome across all observations. Unadjusted estimate is from an unweighted logit model with no additional variables. MSM adjusted for both time invariant (gender, education, ethnicity) and time-varying confounders (age, age squared, employment status [current and one year lagged], benefit status [lagged], home ownership status [lagged], marital status [lagged], number of children [lagged], government office region [lagged], SF-12 physical health component [lagged], SF-12 mental health component [lagged], and previous indication of common mental disorder [one-year lagged GHQ-12 caseness]). Adjusted FE model includes time-varying confounders. Absolute risk difference indicates %-point change. Reference group for transition into poverty is those not in poverty in both years; reference group for transition out of poverty is those in poverty in both years.

Table S5: Causal effect estimates of poverty status on likelihood of common mental disorder (complete cases compared with imputation sample)

	Complete cases: Odds ratio	Imputed results (Table 1): Odds ratio	Complete cases: %-point change	Imputed results (Table 1): %-point change
**Unadjusted estimate**	1.80 (1.73, 1.88)	1.73 (1.67, 1.79)	9.97% (9.20%, 10.73%)	9.45% (8.83%, 10.08%)
**Fixed-effects estimate**	1.29 (1.21, 1.37)	1.22 (1.16, 1.29)	6.28% (4.68%, 7.87%)	5.02% (3.75%, 6.28%)
**Fixed-effects estimate, adj.**	1.23 (1.15, 1.31)	1.16 (1.10, 1.23)	4.09% (1.73%, 6.45%)	1.49% (0.25%, 2.72%)
**MSM estimate**	1.19 (1.12, 1.27)	1.17 (1.12, 1.24)	2.29% (1.45%, 3.12%)	2.15% (1.45%, 2.84%)
n	32,138 (132,962 obs)	45,497 (202,297 obs)	32,138 (132,962 obs)	45,497 (202,297 obs)
PAF (from MSM)	5.27% (3.38%, 7.16%)	4.78% (3.38%, 6.19%)		

MSM = marginal structural model; PAF = population attributable fraction. Unadjusted estimate is from an unweighted logit model with no additional variables. MSM adjusted for both time invariant (gender, education, ethnicity) and time-varying confounders (age, age squared, employment status [current and one year lagged], benefit status [lagged], home ownership status [lagged], marital status [lagged], number of children [lagged], government office region [lagged], SF-12 physical health component [lagged], SF-12 mental health component [lagged], and previous indication of common mental disorder [one-year lagged GHQ-12 caseness]). Adjusted fixed-effects model includes time-varying confounders. Complete cases are from observations with complete data on variables of interest from a wave and the wave preceding it.

Table S6: Sensitivity analysis estimating causal effects of poverty status on likelihood of common mental disorder in restricted sample present in all UKHLS waves (with and without application of longitudinal survey weights)

	Unweighted: Odds ratio	Weighted: Odds ratio	Unweighted: %-point change	Weighted: %-point change
**Unadjusted estimate**	1.92 (1.81, 2.03)	1.32 (1.21, 1.43)	11.04% (9.92%, 12.15%)	11.71% (10.28%, 13.14%)
**Fixed-effects estimate**	1.25 (1.15, 1.37)	1.20 (1.11, 1.30)	6.83% (4.70%, 8.95%)	5.69% (2.99%, 8.40%)
**Fixed-effects estimate, adj.**	1.95 (1.82, 2.10)	1.26 (1.13, 1.40)	4.43% (1.13%, 7.74%)	3.81% (0.11%, 7.52%)
**MSM estimate**	1.21 (1.12, 1.32)	1.22 (1.11, 1.33)	2.52% (1.40%, 3.64%)	2.61% (1.35%, 3.88%)

Analysis completed only on individuals who participated in all nine waves of UKHLS data collection (item missingness within these observations was imputed). Weights used are the longitudinal survey weights supplied by UKHLS which adjust for initial selection and attrition/non-response. In all columns, n=14,239; obs=73,625. MSM = marginal structural model. Unadjusted estimate is from an unweighted logit model with no additional variables. MSM adjusted for both time invariant (gender, education, ethnicity) and time-varying confounders (age, age squared, employment status [current and one year lagged], benefit status [lagged], home ownership status [lagged], marital status [lagged], number of children [lagged], government office region [lagged], SF-12 physical health component [lagged], SF-12 mental health component [lagged], and previous indication of common mental disorder [one-year lagged GHQ-12 caseness]). Adjusted fixed-effects model includes time-varying confounders.

Figure S1: Flowchart of sample selection and exclusion

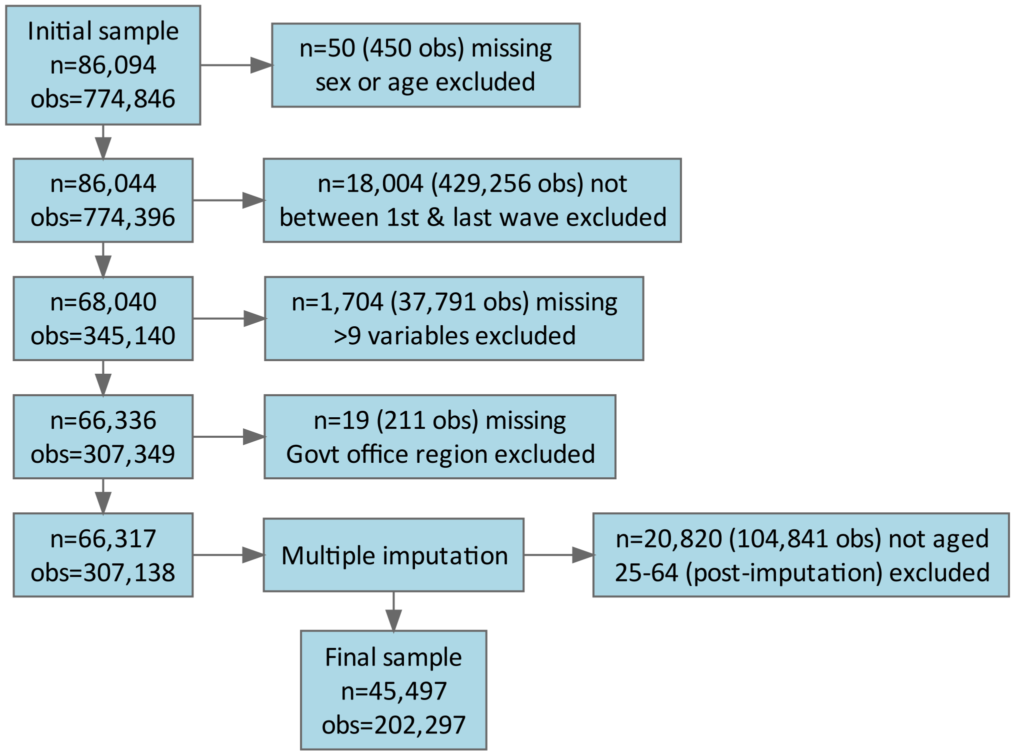

Supplement: dyac226_Supplementary_Data [file dyac226_supplementary_data.pdf]
